# Titanium or Biodegradable Osteosynthesis in Maxillofacial Surgery? In Vitro and In Vivo Performances

**DOI:** 10.3390/polym14142782

**Published:** 2022-07-07

**Authors:** Barzi Gareb, Nico B. Van Bakelen, Arjan Vissink, Ruud R. M. Bos, Baucke Van Minnen

**Affiliations:** Department of Oral and Maxillofacial Surgery, University Medical Centre Groningen, Hanzeplein 1, 9713 GZ, P.O. Box 30001, 9700 RB Groningen, The Netherlands; n.b.van.bakelen@umcg.nl (N.B.V.B.); a.vissink@umcg.nl (A.V.); r.r.m.bos@umcg.nl (R.R.M.B.); b.van.minnen@umcg.nl (B.V.M.)

**Keywords:** biocompatible materials, absorbable implants, polymers, orthopedic fixation devices, reconstructive surgical procedures, fracture fixation

## Abstract

Osteosynthesis systems are used to fixate bone segments in maxillofacial surgery. Titanium osteosynthesis systems are currently the gold standard. However, the disadvantages result in symptomatic removal in up to 40% of cases. Biodegradable osteosynthesis systems, composed of degradable polymers, could reduce the need for removal of osteosynthesis systems while avoiding the aforementioned disadvantages of titanium osteosyntheses. However, disadvantages of biodegradable systems include decreased mechanical properties and possible foreign body reactions. In this review, the literature that focused on the in vitro and in vivo performances of biodegradable and titanium osteosyntheses is discussed. The focus was on factors underlying the favorable clinical outcome of osteosyntheses, including the degradation characteristics of biodegradable osteosyntheses and the host response they elicit. Furthermore, recommendations for clinical usage and future research are given. Based on the available (clinical) evidence, biodegradable copolymeric osteosyntheses are a viable alternative to titanium osteosyntheses when applied to treat maxillofacial trauma, with similar efficacy and significantly lower symptomatic osteosynthesis removal. For orthognathic surgery, biodegradable copolymeric osteosyntheses are a valid alternative to titanium osteosyntheses, but a longer operation time is needed. An osteosynthesis system composed of an amorphous copolymer, preferably using ultrasound welding with well-contoured shapes and sufficient mechanical properties, has the greatest potential as a biocompatible biodegradable copolymeric osteosynthesis system. Future research should focus on surface modifications (e.g., nanogel coatings) and novel biodegradable materials (e.g., magnesium alloys and silk) to address the disadvantages of current osteosynthesis systems.

## 1. Introduction

Most of the osteosynthesis systems applied in oral and maxillofacial surgery (OMF-surgery) consist of plates and screws. In maxillofacial traumatology, osteosynthesis systems are used for fixation of bone segments after anatomical reduction of dislocated or mobile fractures. In orthognathic surgery, they are used for fixation of osteotomy segments in a predetermined position to treat maxillofacial deformities.

Titanium osteosynthesis systems are currently the gold standard for maxillofacial fracture treatment and orthognathic surgery. The titanium plate and screw combination has excellent mechanical and handling properties, providing adequate bone stability with clinically acceptable plate and screw dimensions without the need for rigid maxillomandibular fixation [1,2,3,4,5,6,7]. However, the disadvantages of titanium systems include temperature sensitivity [7], tactile sensation of plates and screws [8], possible growth restrictions [9], hampering of imaging and radiotherapy [10,11,12], presence of titanium particles in surrounding tissue [13,14,15], high elastic modulus causing stress shielding of the underlying bone [12], and potential mutagenicity [7].

The potential of using biodegradable biomaterials to increase health-care quality and reduce costs has led to a substantial increase in interest in biomaterials by researchers as well as clinicians over recent years [16,17,18]. Commercially available biodegradable osteosynthesis systems are commonly composed of synthetic polymers (e.g., polylactide; Table 1). However, in the last decade, researchers have also focused on novel classes of biodegradable materials for osteosynthesis systems such as degradable metals (e.g., magnesium, zinc, iron, and their alloys) and natural polymers (e.g., silk) [18]. Biodegradable osteosynthesis systems composed of synthetic (co)polymers can reduce the need for removal of osteosynthesis systems in a second operation while also avoiding most of the aforementioned disadvantages of titanium osteosyntheses [19]. Biodegradable systems have, however, their own limitations including decreased mechanical properties [20], palpability due to bulkiness [19], and possible foreign body reactions [21].

Over the last few decades, both the titanium and biodegradable osteosynthesis systems have been improved [18,22]. Examples of improvements are the adaption of the production process of titanium systems (e.g., to increase or decrease the elastic modulus of the plates) [23,24], modulating biodegradable polymer compositions (e.g., using L- and D-chirality of lactic acid, or by copolymerization with different homopolymer ratios) [18,20,25], and using ultrasound pin welding of thermoplastic pins for plate fixation instead of using conventional screws [20]. These improvements affect the mechanical properties of the system as well as the host response and, in turn, the complications that arise as a result [18,20]. In addition, recent research provides new insights into long-term host response to biodegradable biomaterials [18,25,26]. Currently, there is no overview that summarizes and discusses these novel insights to guide clinical usage and future research of these osteosynthesis systems.

This review explores advances gathered from in vitro and in vivo studies (i.e., including the clinical performances) on biodegradable and titanium osteosyntheses in maxillofacial surgery. First, the osteosynthesis concepts in maxillofacial surgery are briefly summarized. Next, the factors underlying the favorable clinical outcome of biodegradable osteosyntheses as an alternative to titanium osteosyntheses based on pre-clinical evidence are reviewed, including the physico-chemical and mechanical properties of the (co)polymers as well as the degradation characteristics and the host response they elicit. Then, the clinical outcomes of both types of osteosyntheses are compared and discussed based on the available evidence followed by clinical recommendations. Finally, leads for future research are given and future directions for the clinical translation of novel biomaterials are discussed. Together, these aspects will guide evidence-based selection of osteosynthesis systems in maxillofacial surgery and target future research.

**Table 1:** Commercially available biodegradable osteosynthesis systems for maxillofacial surgery.

Brand Name	Manufacturer	Composition	Indication	Biodegradation Duration	Refs
***Homopolymer (first generation)***					
Biofix SR-PGA	Bionx Implants (Tampere, Finland)	100% SR PGA	Midface and mandible fractures and osteotomies	LM: 36 months	[27,28]
Biofix SR-PLLA	Bionx Implants (Tampere, Finland)	100% SR PLLA	Midface and mandible fractures and osteotomies	LM: >54 months	[27,28]
FIXORB-MX	Teijin Medical Technologies Co., Ltd. (Osaka, Japan)	100% PLLA	Midface and mandible fractures and osteotomies	LM: >3 years	[22]
GrandFix	Gunze (Kyoto, Japan)	100% PLLA	Midface and mandible fractures and osteotomies	LM: >3 years	[22,29,30,31]
***Copolymer (second generation)***					
BioSorb FX	ConMed Linvatec Biomaterials Ltd. (Tampere, Finland)	70% SR PLLA, 30% SR PDLLA	Midface fractures and osteotomies, and mandibular symphysis factures	SEM with EDX: >4 years	[20,25]
Delta	Stryker (Kalamazoo, MI, USA)	85% PLLA, 10% PGA, 5% PDLA	Midface fractures and osteotomies	Visual inspection: 8–13 months	[18,32]
Inion CPS	Inion Oy (Tampere, Finland)	70–78.5% PLLA, 16–24% PDLLA, 4% TMC ^1^	Midface and mandible fractures and osteotomies	SEM with EDX: >4 years	[20,25]
Inion CPS Baby	Inion Oy (Tampere, Finland)	82% PLLA, 12% PGA, 6% TMC	Cranial reconstructions, including midface and mandibular fracture fixation, in pediatric patients	Ultrasonography: 2–3 years	[33,34]
LactoSorb	Biomet Microfixation (Jacksonville, FL, USA)	82% PLLA, 18% PGA	Midface fractures and osteotomies	SEM with EDX: >4 years	[18,20,25]
Macropore	Medtronic, Inc. (Minneapolis, MN, USA)	70% PLLA, 30% PDLLA	Midface fractures and osteotomies	Unknown	[20]
MacroSorb	Medtronic, Inc. (Minneapolis, MN, USA)	70% PLLA, 30% PDLLA	Midface and mandible fractures and osteotomies	LM: >12 months	[27,35]
Polymax	Synthes (Oberdorf, Switzerland)	70% PLLA, 30% PDLLA	Midface and mandible fractures and osteotomies	LM: >12 months	[20,27,35]
Polymax RAPID	Synthes (Oberdorf, Switzerland)	85% PLLA, 15% PGA	Midface and mandible fractures and osteotomies	Unknown	[27]
RapidSorb	DePuy Synthes (West Chester, PA, USA)	70% PLLA, 30% PDLLA	Midface fractures and osteotomies	In vitro: 12 months	[20,22]
Resomer	Evonik Industries (Darmstad, Germany)	50% PLLA, 50% PDLLA	Midface fractures and osteotomies	Unknown	[27]
ResorbX	KLS Martin Group (Gebrüder Martin GmbH & Co., Tuttlingen, Germany)	100% PDLLA	Midface fractures and osteotomies	LM: 12–30 months	[18,20]
SonicWeld Rx	KLS Martin Group (Gebrüder Martin GmbH & Co., Tuttlingen, Germany)	100% PDLLA	Midface fractures and osteotomies	SEM with EDX: >4 years	[20,25]
SonicWeld xG	KLS Martin Group (Gebrüder Martin GmbH & Co., Tuttlingen, Germany)	85% PLLA, 15% PGA	Midface fractures and osteotomies	LM: 12–14 months	[18,20]
***Biocomposite (third generation)***					
OsteotransMX	Teijin Medical Technologies Co., Ltd. (Osaka, Japan)	Plate: 60% PLLA, 40% uHA Screw: 70% PLLA, 30% uHA	Midface and mandible fractures and osteotomies	LM: 5.5 years	[20,22,36,37]

^1^ The manufacturer does not publicly report the exact composition of the copolymers. PLLA, poly-L-lactic acid; PDLLA, poly-D,L-lactic acid; TMC, trimethylene carbonate; SR: self-reinforced; PGA, poly-glycolic acid; uHA, unsintered hydroxyapatite; LM, light microscopy; SEM, scanning electron microscopy; EDX, energy-dispersive X-ray analysis.

## 2. Pre-Clinical Evidence

A biodegradable osteosynthesis system should meet two intertwined criteria to be used as an osteosynthesis system: (1) the biomaterial needs to be biocompatible with the host tissue and (2) the mechanical properties should be sufficient for stable fixation of fracture or osteotomy segments during the surgical procedure (primary stability) and during the degradation of the biomaterial, with a gradual transfer of stress to the healing bone [18].

### 2.1. Biocompatibility

#### 2.1.1. Initial Host Response

Implanted materials evoke an initial host response after implantation that includes inflammation, proliferation and tissue remodeling, and, in the case of biodegradable biomaterials, is affected by the degradation products [18]. This host response is mediated by both the innate and adaptive immune systems. Macrophages are the most important innate immune cells during the host response and also play a main role in the outcome of biodegradable implants [18]. The phenotype of macrophages ranges from pro-inflammatory M1 macrophages to anti-inflammatory M2 macrophages [38,39]. After tissue injury, M1 macrophages secrete several inflammatory mediators such as interleukin-1 (IL-1) and tumor necrosis factor-α (TNF-α) to initiate the healing process [18,40]. After the initial inflammatory phase, macrophages switch to a wound-healing phenotype (M2a), secreting growth factors (e.g., platelet-derived growth factor) that promote angiogenesis and cell proliferation [40,41]. Subsequently, macrophages switch to an anti-inflammatory phenotype (M2c) and produce anti-inflammatory cytokines (e.g., IL-10) that leads to the inhibition of the inflammatory response [42].

The adaptive immune system is also involved in the host response. Through antigen presentation, macrophages and dendritic cells can activate CD4^+^ T-cells of the adaptive immune system. T helper 1 (T_H_1) cells can induce M1 macrophages by producing interferon-γ and IL-2 [43]. Subsequently, M1 macrophages can produce cytokines and chemokines (e.g., IL-12, CXC-chemokine ligand 9) that intensify the T_H_1 response by recruiting additional T_H_1 cells [18]. In contrast to T_H_1 cells, T_H_2 cells produce anti-inflammatory cytokines (e.g., IL-4 and IL-10) that induce polarization of macrophages towards M2 macrophages. M2 macrophages in turn secrete cytokines (e.g., CC-chemokine ligand 17) that recruit additional T_H_2 cells that temper the inflammatory response [43]. Imbalances of M1 over M2 macrophages or prominent presence of M1 macrophages may lead to (chronic) foreign body reactions (e.g., a sterile abscess formation with fibrous encapsulation) [18]. Therefore, it is essential that a well-controlled and timely switch of M1 to M2 macrophages occurs as this then leads to implant degradation and tissue remodeling, to eventually replace the implant by host tissue (biodegradable systems) or to controlled fibrous encapsulation (titanium systems) [18].

#### 2.1.2. Synthetic Biodegradable Polymers

The most commonly used (co)polymers in biodegradable osteosynthesis systems consist of poly(α-esters) such as poly(L-lactic acid) (PLLA), poly(D,L-lactic acid) (PDLLA), poly(lactic-*co*-glycolic acid) (PLGA), or poly(L-*co*-D,L-lactic acid-*co*-trimethylene carbonate) (P(LLA-co-DLLA-co-TMC)) (Table 1) [18].

##### Biodegradation

Synthetic polymers undergo biodegradation via two different modes depending on the rates of bond cleavages and water diffusion into the polymer: bulk and surface degradation. In bulk degradation, the degradation occurs in the complete implant resulting in a decrease in molecular weight and molecular strength with time. Since the complete implant degrades at a similar rate, disintegration of the implant with generation of polymeric debris can occur. In contrast, surface degradation occurs on the surface of the implant, resulting in a decrease in size and mass of the implant with time. Here, the molecular weight and mechanical properties of the material remain relatively unchanged [18].

Extracellular degradation of poly(α-esters) occurs through hydrolysis (two phases), enzymatic degradation, and oxidation. During hydrolysis, cleavage of the ester bonds by water results in oligomers and monomers, such as lactic acid and glycolic acid (primary hydrolysis) [44,45], that can enter the tricarboxylic-acid cycle (secondary hydrolysis) to form carbon dioxide and water that can be excreted in the lungs or via urine. Secondary hydrolysis is the rate-limiting step and depends highly on the crystallinity and hydrophobicity of the intermediate products [16]. Enzymes secreted by macrophages and derived from the blood can contribute to hydrolysis through extracellular hydrolysis [18]. Macrophages can also phagocytize biomaterial particles. In addition, inflammatory cells (e.g., macrophages and neutrophils) can induce depolymerization of polymers by oxidation via the release of reactive oxygen species [46]. Macrophages can also undergo fusion to improve their efficiency and form multinucleated giant cells [47] which can remain for up to 24 months after implantation [25]. Although the phagocytosis capacity of multinucleated giant cells is reduced compared to macrophages, the capacity of extracellular degradation is increased by secreting higher concentrations of enzymes and reactive oxygen species into the interface between the multinucleated giant cells and implant [47].

##### Late Host Response

Biodegradable osteosynthesis systems should, preferably, be completely resorbed within 12 months [17]. However, foreign body reactions to polymeric biodegradable materials remain a major concern, even years after implantation [26]. Factors that influence foreign-body reactions are implant related (e.g., polymer composition, crystallinity, geometry, and surface topology), recipient related (e.g., blood supply), and plate location related (e.g., epiperiosteal versus subperiosteal) [18,48,49].

The progression of the host response is affected by the acidic degradation products of the poly(α-esters). A lowering in pH intensifies the inflammatory response that results in fibrous encapsulation of the implant [50,51]. Furthermore, the acidic degradation products are autocatalytic, resulting in progressive degradation of the remaining polymers and an increase in the inflammatory response. Additionally, bulk degradation leads to fragmentation of the polymer that may result in phagocytized particles within the fibrous tissue [18]. Demineralization of the surrounding bone can occur whenever the degradation occurs too quickly and the surrounding tissue fails to eliminate the degradation products [52]. Therefore, the possibility to induce a foreign body reaction is dependent on an equilibrium between the levels of degradation products, the degree of fibrous encapsulation, and the ability of the host to eliminate the degradation products [18]. Short-term foreign body reactions are mainly caused by fast-degrading polymers (e.g., PGA) [53] while delayed foreign body reactions are often associated with slow-degrading polymers (e.g., PLLA) with high crystallinity and crystalline degradation fragments [21,54,55]. Foreign body reactions to polymeric biodegradable materials can occur to particle sizes of <2 µm, even years after the implantation (Table 2) [26].

Currently, two main hypotheses regarding the etiology of foreign body reactions to these synthetic polymeric biomaterials exist. After implantation, the biodegradable polymers are encapsulated by fibrous tissue that acts as a semi-permeable membrane [48]. The first hypothesis is that, as the polymer degradation continues over time, the size of the polymeric fragments decreases while the number of particles increases. These particles cannot pass the semi-permeable membrane. Subsequently, the osmotic pressure within the area surrounded by the fibrous layer increases and this results in a clinically observable swelling that, without an intervention, remains [21]. An alternative hypothesis is that, eventually, the acidic polymeric fragments become small enough to pass the membrane. This results in a decrease in pH of the surrounding tissues which then causes excessive sterile inflammation [56,57] accompanied by phagocytosis of any residual fragments [48]. However, since crystalline fragments are stable and more resistant to further hydrolytic degradation, they accumulate in the macrophages and multinucleated giant cells, and then remain in situ. Furthermore, extra- and intracellular residual fragments can lead to the accumulation of crystalline oligomeric stereo-complexes over time that are resistant to further hydrolytic degradation [18,58]. These two hypotheses could also occur simultaneously.

Differences in vascularization also contribute to inducing foreign body reactions. Sufficient vascularization is necessary for adequate bone healing, but it is also essential to eliminate the acidic degradation products of the hydrolyzed poly-α-esters (e.g., polylactide), thereby affecting the equilibrium between the levels of degradation products and the ability of the host to eliminate the degradation products [18]. Accumulation of acidic degradation products may result in decreased pH [18], bone demineralization [52], and may damage the surrounding cells such as macrophages [59,60,61]. Whenever micromovements are present, fibrous encapsulation can entrap the acidic degradation products, resulting in reduced elimination of the degradation products [18]. The acidic degradation products have an autocatalytic effect and cause further degradation of the remaining polymer resulting in a vicious circle that eventually leads to a more severe inflammatory reaction [18]. Since the mandible has lesser vascularization and is exposed to higher forces, mandibular osteosyntheses are more prone to these (accumulating) effects compared to those in other parts of the facial skeleton.

**Figure 1:**
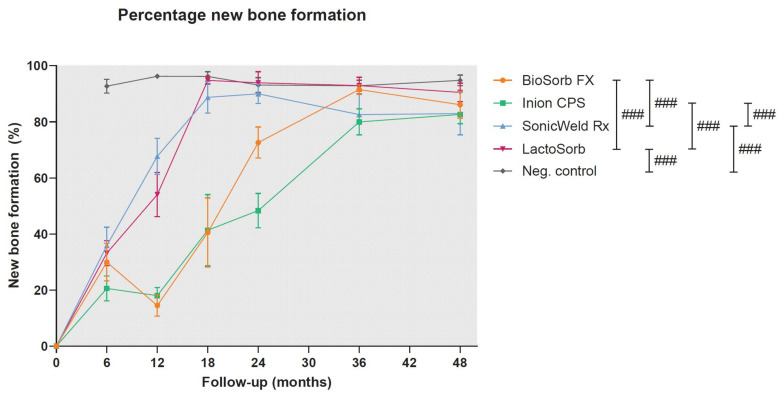
Percentage of total new bone formation at the implant site. Error bars: mean values ± standard error of the mean. ### represents *p* < 0.05, *p* < 0.01, and *p* < 0.001, respectively. The composition of each system is described in Table 1. A similar curve for titanium systems is not applicable due to the non-degradable nature of titanium systems. Error bars: mean values ± standard error of the mean. Reprinted with permission from [25].

**Figure 2:**
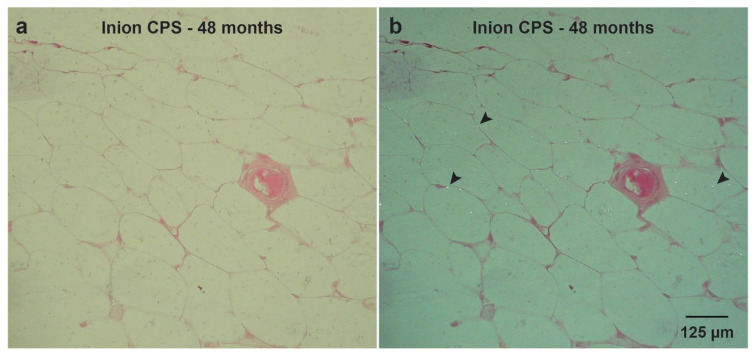
HE-sections of the Inion CPS system at 48-month follow-up under LM (**a**) and LM-pol (**b**) with observable residual polymer fragments (examples are indicated with black arrows). HE, hematoxylin and eosin; LM, light microscopy; LM-pol, polarized light microscopy. From the study of [25].

**Figure 3:**
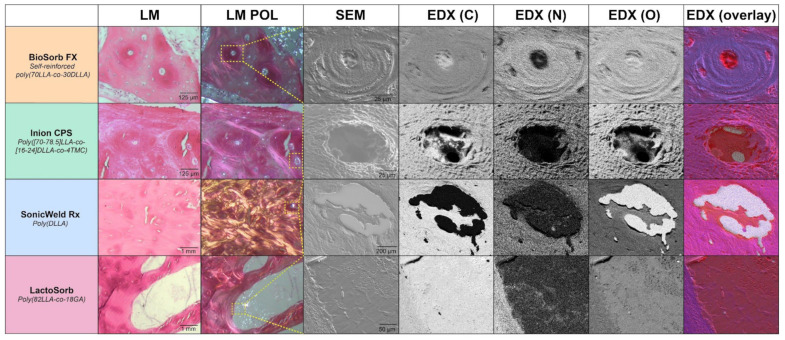
HE-sections (LM and LM-pol), SEM, and EDX by element and with overlay (red: carbon, and blue: nitrogen) of birefringent polymeric residual fragments of every osteosynthesis system at 48-month follow-up. HE, hematoxylin and eosin; LM, light microscopy; LM-pol, polarized light microscopy; SEM, scanning electron microscopy; EDX, energy-dispersive X-ray analysis; C, carbon; N, nitrogen; O, oxygen. Reprinted with permission from [25].

In a recent study, the long-term (i.e., up to 4-year follow-up) biocompatibility and degradation of four commonly used biodegradable copolymeric osteosynthesis systems was compared using a goat model [25]. The study included the BioSorb FX [poly(70LLA-*co*-30DLLA)], Inion CPS [poly([70–78.5]LLA-*co*-[16–24]DLLA-*co*-4TMC)], SonicWeld Rx [poly(DLLA)], and LactoSorb [poly(82LLA-*co*-18GA)] biodegradable osteosynthesis systems. The copolymer of the SonicWeld Rx system was the only one that was amorphous; all the other assessed systems were semi-crystalline. All the biodegradable systems were safe to use and well-tolerated. The SonicWeld Rx system showed the most predictable degradation profile. In addition, together with the LactoSorb system, new bone percentages similar to negative controls were observed after 18 months while the two other included systems reached these levels after 36 months (Figure 1). However, nanoscale residual polymeric fragments, predominately accumulated in adipocytes (Figure 2), were observed at every system’s assessment (Figure 3).

Since the crystalline regions of synthetic (co)polymers, the intermediate degradation products and the crystalline oligomeric stereo-complexes that can be formed in vivo over time are hydrophobic [17,58,62], this could explain the remarkable accumulation of polymeric birefringent fragments in adipocytes within the medullary bone cavity up to 4-year follow-up (Figure 2) [25]. Similar birefringent fragments, derived from as-polymerized PLLA, were observed in a case report [21] and experimental studies up to the 5-year follow-up [63,64]. Such particles were found intracellular after 3 and 4.5 years of implantation, although the particles decreased in size over time [63]. Crystalline fragments derived from as-polymerized PLLA can induce foreign body reactions even up to 5.7 years after implantation [21,65]. Another clinical study that focused on the efficacy of an osteosynthesis system composed of unsintered hydroxyapatite/PLLA composite, with a 12-month follow-up, showed that the removed symptomatic systems included up to 65% crystalline regions in the explanted polymers [66]. In a study that implanted the Resorb X osteosynthesis system (PDLLA) at the condyle of sheep mandibles, no foreign body reactions and complete bone formation were observed after 12 months [67]. Another study showed complete bone formation 18 months after implanting the LactoSorb system in the maxillofacial area of Göttingen minipigs without signs of foreign body reactions [68]. In contrast, after implanting the Inion CPS system in sheep, the system was surrounded by a fibrous capsule with granulomatous foreign body reactions after 52 weeks [69]. In the literature, foreign body reactions have predominately been reported for biodegradable osteosyntheses with a high proportion (i.e., >70%) of PLLA [18,21,55,70] or poly(glycolic acid) (PGA) [18]. More amorphous copolymers such as PDLLA (e.g., 50LLA/50DLA ratio) are more hydrophilic, and degrade and resorb more quickly and predictably [71]. These findings, as well as those of different (pre-)clinical studies [18,25,26,72], emphasize that the (co)polymers used in biodegradable systems should be completely amorphous. Future research should focus on amorphous (co)polymers with a minimum follow-up of ≥24 months so that a proper degradation assessment can be performed. Furthermore, it remains unknown whether the observed nanoparticles after 4-year follow-up [25] may be harmful in the long run (i.e., >4 years). Since microplastics have been shown to be toxic in vitro, with a potential impact on human health (e.g., effects on the gastrointestinal tract, lungs, immune system, and blood components) [73,74], the effects of the observed nanoparticles need further research.

Other than (co)polymer composition, the geometry and surface topography of the implanted materials also affect biocompatibility in vivo [72]. Thick biomaterials, especially with points and sharp edges, can increase the risk of foreign body reactions [26,75,76]. In contrast, thinner biomaterials, as well as smaller sized polymeric particles used to engineer a biomaterial, allow for quicker degradation and a lower risk of foreign body reactions [72,77,78]. A smooth well-contoured shape without acute angles induced macrophage polarization towards macrophages with an immune regulatory phenotype [79,80]. In vivo biocompatibility of medical devices, such as implants, can be significantly improved by tuning the spherical dimensions [72]. Furthermore, low implant volume reduces the amount of acidic degradation products and thus reduces the risk of (late) foreign body reactions [18]. The fact that screws possess acute angles, while welded pins do not, may explain the favorable degradation profile of the SonicWeld Rx system compared to the BioSorb FX, Inion CPS and LactoSorb biodegradable systems [18,25,81,82]. Novel biodegradable system development should incorporate geometry and surface topography into the design-phase as these characteristics are tunable and may be efficient ways to decrease foreign body reaction risk, hasten degradation, enhance quicker bone formation, and balance the degradation and regeneration equilibrium (Table 2) [26].

**Table 2:** Different aspects of biodegradable osteosynthesis systems accompanied with the ideal properties and the potential solutions to accomplish these properties.

Aspect	Ideal Properties	Method	Potential Solutions	Refs
**Surgical handling**	Easy perioperative adaptation of plates	3D engineering	Patient specific osteosynthesis systems	[18,26,83]
		Production process	Plate adaption at room temperature	[20]
	No risk of perioperative screw breakage	Alternative application method	Ultrasound welding of thermoplastic pins instead of using conventional screws	[20]
**Elastic modulus of materials**	Enough elastic modulus to avoid micromovements, but not stiffer than bone to avoid stress-shielding of the underlying bone	Production process	Create composites to tailor the elastic modulus to the application of interest	[26]
			Self-reinforcing of polymers to increase the elastic modulus of systems	[20]
		Alternative application method	Ultrasound welding of thermoplastic pins to increase the maximum tensile load and stiffness, and side-bending stiffness	[20]
**Bacterial infection**	Preventing bacterial adhesion to implant surface	Coating	Hydrophobic coatings	[26]
	Eliminating surrounding bacteria without antibiotics	Surface modification	Adjusting the nano-scale surface topography (e.g., pillars on the surface)	[84]
	Eliminating surrounding bacteria with local antibiotics		Polymer coating containing stabilized gas bubbles loaded with antibiotics that can be released locally using ultrasound	[85]
**Foreign body response (FBR)**	Materials that do not elicit an FBR	Selection of materials	Materials with non-toxic degradation products (e.g., derived from silk)	[18]
		Production process	Avoid thick materials, especially with points and sharp edges	[26,75,76]
	Tailor the host response so that FBR are avoided	Production process	Avoid particle sizes < 2 µm	[26]
	Avoid micromovements (max. 28–150 µm), that can result in fibrous encapsulation of the implant	Selection of materials, production process, and 3D engineering	Osteosynthesis system with material properties that matches with the mechanical properties of the target tissue (e.g., by using ultrasound welding)	[26]
**Degradation profile**	Predictable degradation, preferably after 3–12 months	3D engineering	Thinner materials degrade quicker	[17]
		Production process	Balance the degradation and regeneration equilibrium by, e.g., using L- and D-chirality or by copolymerization	[25,26]

#### 2.1.3. Biodegradable Metals

Biodegradable metals are promising alternatives to polymeric osteosynthesis systems due to their mechanical properties that are closer to bone than (co)polymeric materials [18] and their less harmful degradation products. The tensile strength, elastic modulus, axial pull-out force, and maximum torque of magnesium alloys are higher than that of (co)polymers, but lower than that of titanium alloys [18,86]. To date, three biodegradable metal groups have been researched to be used for biodegradable osteosynthesis systems, i.e., magnesium (Mg), iron (Fe), and zinc (Zn) and their alloys [18]. Mg-based biodegradable metals have been studied most extensively. The available research for Fe- and Zn-based degradable metals is limited due to the low degradation rate of Fe-based metals while Zn-based metals have been introduced only recently [18].

##### Biodegradation

Biodegradable metal degradation is driven by anodic and cathodic reactions that result in the production of oxides, hydroxides and/or hydrogen gas [18,87]. Once biodegradable metals come into contact with body fluids, they are oxidized into metal cations combined with producing electrons via an anodic reaction. The electrons generated by implanting Mg-based biodegradable metals are consumed by cathodic reactions with water to form hydrogen gas and hydroxide. For Fe- and Zn-based metals, oxygen reduction only produces hydroxide without hydrogen gas. Hydroxide then reacts with the adjacent metal to form a metal-hydroxide layer on the surface of the implant. The protective layer can be eroded by high levels of chloride ions in the body fluids resulting in continuation of the degradation process. However, in Fe-based biodegradable metals, the protective layer consists of Fe(OH)_2_, Fe(OH)_3_, and Fe_3_O_4_, that inhibits further degradation. As a result, the degradation rate of Fe-based metals is very slow [18]. These ongoing reactions cause an oversaturation of calcium and phosphate ions in the surrounding body fluids that result in a layer of calcium-phosphate on the metal-oxide layer, that is able to induce bone formation [18].

A major challenge of biodegradable metals, particularly Mg-based materials, is the unpredictable degradation profile in vivo with subcutaneous emphysema due to the accumulation of hydrogen gas [18]. The degradation rate of biodegradable metals can be controlled by tailoring the microstructure, surface properties and coatings of the materials. For example, a recent study included gallium (i.e., a bone resorption inhibitor) in a magnesium alloy and showed promising results with inhibition of bone porosity formation, mechanical properties matching cortical bone, and low corrosion rate resulting in less hydrogen gas formation compared to other available magnesium alloys for orthopedic surgery [88]. In addition, surface modifications and coatings can be used to tune the degradation rates. For example, Mg-alloys and polymers can be combined to form Mg–polymer composites. These composites include high strength and elastic modulus derived from biodegradable metals while the surrounding biodegradable polymer matrix improves the corrosion resistance of the underlying metal [89].

##### Late Host Response

The degradation products of degradable metals such as hydroxide ions, hydrogen gas, metal-oxides, abraded particles, and calcium-phosphate affect the host response [90,91]. In a bone environment, the formation of the calcium-phosphate layer induces new bone deposition, making it a unique feature as base material for an osteosynthesis system. In addition, the Mg-ions can induce new bone formation in cortical bone by increasing calcitonin gene-related peptide 1 levels in periosteum-derived stem cells [92]. However, current Mg-based biodegradable metals often show a burst release of Mg-ions that can lead to excess formation of hydrogen gas resulting in gas pockets, tissue displacement, and subcutaneous emphysema. The fast degradation rate can also induce osteolysis, hemolysis, and rapid reduction of the mechanical properties [93].

#### 2.1.4. Silk

Silk is the most recent addition to biodegradable materials [18]. Silk is a natural biodegradable polymer that is usually derived from the silkworm *Bombyx mori*. Although the evidence is still limited to pre-clinical evidence, the current evidence shows excellent biocompatibility and unique mechanical properties combined with easily and environmentally friendly processing into mechanically robust three-dimensional bulk materials with excellent machinability [18,94]. To date, it is the only natural polymer that has been used to prepare an osteosynthesis system [94].

##### Biodegradation

As with most natural polymers, silk is degraded enzymatically, e.g., by protease XIV, matrix metalloproteinase and collagenase [44]. These enzymes cleave silk protein chains into peptide fragments with decreased molecular weight and strength [95]. Immune cells, especially macrophages and FBGCs, play an important role during degradation of silk. Immune cells mediate silk degradation through (1) phagocytosis and (2) extracellular degradation mediated by proteolytic enzymes derived from macrophages and FBGCs. The degradation products are tightly packed aggregates or amino acids for metabolism [18]. The degradation time depends on implant-related factors (e.g., molecular weight, porosity, crystallinity, and surface topography) and host-related factors (e.g., species and implantation site). The degradation times can be tailored from minutes to years by controlling the material variables such as molecular weight, surface topography, β-sheet content, and porosity [18]. Although in vivo research in animal studies showed complete silk degradation, a thorough understanding of the degradation pathways and clearing mechanisms as well as degradation in humans is still lacking [18].

##### Late Host Response

After implantation of silk materials, a mild inflammatory response occurs that decreases within a few weeks. This host response involves recruitment and activation of macrophages and the formation of FBGCs. The silk implant can be degraded and replaced by host tissue (e.g., bone), but it can also be integrated within the tissue or encapsulated by fibrous tissue. There is currently limited data regarding the short- and long-term host response in vivo. In the currently only available study that prepared a silk-based osteosynthesis systems for fracture fixation in maxillofacial surgery, the in vivo assessment of the 4- and 8-week host response by rats showed more favorable mechanical properties than biodegradable synthetic polymers and excellent biocompatibility accompanied with bone remodeling [94]. These results are promising but additional research is necessary to unravel the complete degradation pathways as well as the host responses that this natural-derived polymer elicits.

#### 2.1.5. Titanium and Its Alloys

##### Late Host Response

Titanium osteosynthesis systems are commonly made of pure titanium or titanium alloys [20,96]. The most frequently used titanium alloy for maxillofacial osteosynthesis systems consists of 90% titanium, 6% aluminum, and 4% vanadium (Ti6Al4V, also called titanium alloy grade 5) [96,97,98,99]. However, although titanium and its alloys are presumed to be completely bioinert, there is growing evidence that wearing of particles occurs that can accumulate in surrounding tissues and different organs of which the consequences are still largely unknown [96,98,100].

**Figure 4:**
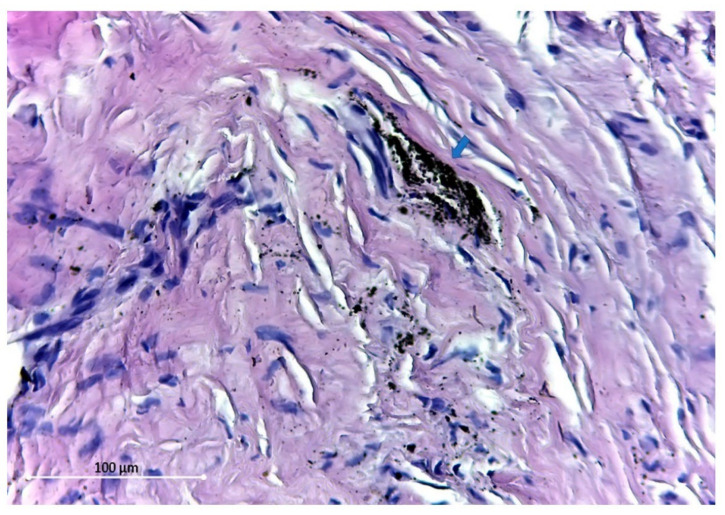
HE-section of a soft tissue biopsy surrounding commercially pure titanium plates after mandibular osteosynthesis under LM. Dust-like (1 micron) particles are indicated with a blue arrow (magnification ×400). HE, hematoxylin and eosin; LM, light microscopy. Reprinted with permission from [15].

In a study that explanted titanium osteosynthesis plates from patients that underwent craniofacial surgery, titanium particles (7.9 to 31.8 µg/gram of dry tissue) could be detected in the regional soft tissue and lymph nodes after 24-month follow-up [13]. Similarly, a recent study showed that the tissue surrounding titanium plates after fracture and osteotomy fixation contained 1.03 and 1.09 ppm titanium particles, respectively [14]. Meningaud et al. revealed a large variation in titanium levels within the surrounding tissue (4–8000 µg/gram) after titanium fixation of osteotomies, but concluded that almost all of these particles were produced at the moment of applying the osteosynthesis system [101]. Other studies reported on the presence of dark-grey pigmentation accompanied with fibrosis of the surrounding tissue and macrophages containing intra-cellular titanium particles (Figure 4) [15,102,103]. Zaffe et al. have also shown the presence of titanium in the surrounding tissue as well as that erythrocytes and lymphocytes contained titanium particles [104]. In addition, explanted osteosynthesis plates analyzed with scanning electron microscopy showed defects and irregularities most likely due to in vivo substance loss [102]. Titanium debris has also been found throughout the body suggesting hematogenous dissemination, with traceable amounts of titanium particles within the liver, spleen, and lymphatic system [98,100].

**Figure 5:**
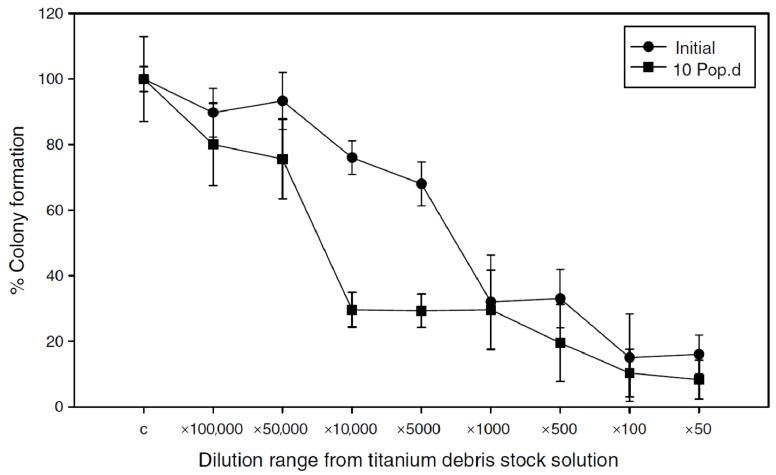
The initial curve represents the percentage survival of colony formation of HF19 cells exposed to titanium particles for 24 h. The percentage of survival of the progeny of these cells is also shown, indicating delayed reproductive death 10 generations postexposure. All percentages are expressed relative to the control expressed as 100%. Reprinted with permission from [105].

To determine the effect of such titanium particles, Coen et al. assessed the cytotoxicity of Ti6Al4V particles on human fibroblast cells in vitro, and showed chromosomal instability, reproductive failure and decreased clonogenic survival 10 generations postexposure (Figure 5) [105]. Studies that analyzed the periosteum surrounding titanium plates as well as blood samples in patients after mandibular fracture fixation showed redox abnormalities, and increased oxidative stress and damage [106,107]. Furthermore, an association between aluminum and the pathogenesis of Alzheimer’s disease has been suggested. In addition, increased levels of circulating aluminum are associated with microcytic anemia and osteomalacia [98,108,109]. These findings indicated that there is a need for long-term epidemiological studies that assess the effect of these particles in the long run.

**Figure 6:**
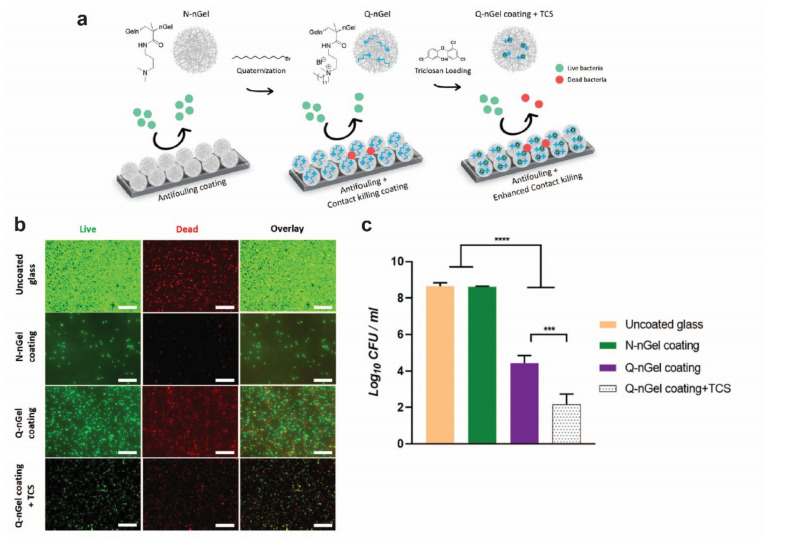
(**a**) Schematic overview of nanogel coating with antifouling and antimicrobial properties. (**b**) *Staphylococcus aureus* adhered to glass with and without the nanogel coatings under fluorescence microscopy (scale bars 20 µm). (**c**) The number of colony-forming units of surviving *Staphylococcus aureus* after 24 h incubation on the surface of uncoated and coated glass (*** and **** indicate *p* < 0.001 and *p* < 0.0001, respectively). N-nGel, nonquaternized nanogel; Q-nGel, quaternized nanogel; TCS, triclosan; CFU, colony-forming units. Reprinted with permission from [122].

Surface modifications (e.g., oxygen plasma immersion ion implantation) have been proposed to reduce metal ion release from the implant (Table 3) [110]. In addition, they are an important aspect of biocompatibility [98,110]. Titanium, without surface modifications, has a positively charged surface and will, therefore, tend to covalently bond to negatively charged proteins such as fibronectin [111]. Fibronectin promotes bacterial adhesion and, thus, increases the risk of infection [112]. Besides bonding to autologous proteins, most of the cell surface of bacterial species (e.g., *Staphylococcus aureus*, the most common etiological pathogen of infections surrounding osteosyntheses [113]) is negatively charged, and thus also adheres to positively charged surfaces such as titanium [114]. By modifying the surface charge, adhesion of various bacteria (e.g., *Staphylococcus aureus* and *Escherichia coli*) is inhibited and, ideally, the risk of infection is reduced [110]. These properties of titanium systems can also be tuned by other surface modifications (Table 3 and Figure 6).

**Table 3:** Different aspects of titanium osteosynthesis systems accompanied with the ideal properties and the potential solutions to accomplish these properties.

Aspect	Ideal Properties	Methods	Potential Solutions	Refs
**Surgical handling**	Easy perioperative adaptation of plates	3D engineering	Patient specific osteosynthesis systems	[115,116]
		Production process	Adaption of the production process to alter the mechanical properties of plates (e.g., lower stiffness)	[20,117,118,119,120]
	No risk of perioperative screw breakage	3D engineering	Adjusting the screw head to improve the grip on the screws	[20]
**Elastic modulus**	Enough elastic modulus to avoid micromovements, but not stiffer than bone to avoid stress-shielding of the underlying bone	Production process	Adaption of the production process to alter the mechanical properties of plates	[20,117,118,119,120]
**Bacterial infection**	Preventing bacterial adhesion to implant surface	Coating	Hydrophobic coatings	[26]
			(Nano)gel coatings	[121,122]
		Surface modification	Plasma immersion ion implantation (surface modification)	[110,123,124]
			Physical vapor deposition	[125,126]
			Increasing surface energy by acid etching	[127]
	Eliminating surrounding bacteria without antibiotics	Coating	Titanium Nitride (TiN) coating	[128,129]
		Surface modification	Adjusting the nano-scale surface topography (e.g., pillars on the surface)	[84]
			Plasma immersion ion implantation	[110,130]
			Physical vapor deposition	[131]
			Laser surface modification	[132]
			Anodization	[133,134]
			Micro-Arc oxidation	[135,136]
	Eliminating surrounding bacteria with local antibiotics	Coating	Polymer coating containing stabilized gas bubbles loaded with antibiotics that can be released locally using ultrasound	[85]
			(Nano)gel coatings	[122,137]
		Surface modification	Chemical vapor deposition	[138]
**Osteogenesis**	Improving bone growth surrounding the implant	Coating	(Nano)gel coatings	[137]
		Surface modification	Plasma spraying with hydroxyapatite	[139,140,141,142,143]
			Plasma immersion ion implantation	[144,145]
			Physical vapor deposition	[146,147]
			Chemical vapor deposition	[148]
			Increasing surface energy by acid etching	[127]
			Laser surface modification	[132,149,150]
			Anodization	[151]
**Wear resistance**	No wearing of titanium (alloy) particles	Coating	Titanium Nitride (TiN) coating	[152,153]
		Surface modification	Plasma immersion ion implantation	[110]
			Physical vapor deposition	[98]
			Laser surface modification	[150,154]
			Anodization	[134,155,156]

### 2.2. Mechanical Properties

#### 2.2.1. Minimally Required Mechanical Properties

Several studies assessed the mechanical forces surrounding osteosyntheses applied to maxillofacial fractures [157,158,159,160,161,162,163], osteotomies [164,165] and reconstructions [166], so that the minimally required mechanical properties of an osteosynthesis system can be estimated. After maxillofacial trauma, the reported bite force increases up to 64 N by the second postoperative fracture fixation day, 92 N after 1 week, 187 N after 4 weeks, and up to 373 N at the 3-month follow-up [157]. Other studies focusing on trauma patients showed that 100 N forces were measured after 4 weeks of fixation [159,161]. The mechanical forces around maxillofacial osteotomies have been reported to increase from 21 ± 14 N (i.e., after 1 week) to 65 ± 43 N (i.e., after 6 weeks) [160] while other studies reported forces ranging from 82.5 to 132 N [164,165]. The masticatory forces after mandibular reconstructions ranged from 28 to 186 N [166]. However, the mechanical stress surrounding osteosynthesis systems is multi-factorial and is affected by the location of the fracture [1], differences in interfragmentary stability [1], mandibular height [1], degree and direction of movement [167], and preoperative masticatory forces [159,168,169]. Load-sharing osteosynthesis allows sharing of the load between bone segments and the osteosynthesis system (e.g., fractures with interfragmentary stability) whereas in load-bearing osteosynthesis, the complete load at the fracture site is carried by the osteosynthesis system without interfragmentary stability [1,170]. In a load-bearing situation, the osteosynthesis system is exposed to substantially higher loads and, thus, the biomechanical requirements for an optimal osteosynthesis system are higher compared to load-sharing osteosyntheses [70,171]. Although it would be of high clinical value to determine the exact cut-off value of the transition from load-sharing to load-bearing osteosyntheses, this is currently unknown. Since the mandible is exposed to considerably higher biomechanical forces compared to the maxilla [1], load-bearing osteosynthesis of the mandible requires even higher mechanical properties of the used osteosynthesis system compared to load-bearing osteosynthesis of the maxilla or load-sharing osteosynthesis of the mandible [172,173]. Furthermore, as bone healing progresses, the forces will be shared by the osteosynthesis system and the underlying healing bone. Thus, it remains difficult to estimate the least mechanical properties an osteosynthesis system has to meet. Therefore, researchers have mainly focused on relative differences between the available osteosynthesis systems [20].

#### 2.2.2. Mechanical Properties of Osteosynthesis Systems

The mechanical properties of osteosynthesis systems depend on several factors including composition (i.e., titanium (alloys) or (co-)polymers), the production processes (e.g., stamping versus laser cutting of titanium systems) [117,118,174], dimensions, polymer self-reinforcement [175], the application method (i.e., screws or ultrasound welded pins) [176], ageing, and sterilization methods [177,178,179]. The tensile, bending and torsional stiffness of an osteosynthesis system are a more clinically relevant outcome than maximum tensile load since this affects adequate fixation and bone healing (i.e., malunion and non-union) [180] while maximum tensile load is only relevant whenever the bone segments are already separated by more than a few millimeters. In the latter case, this will certainly result in compromised bone healing or malunion.

**Figure 7:**
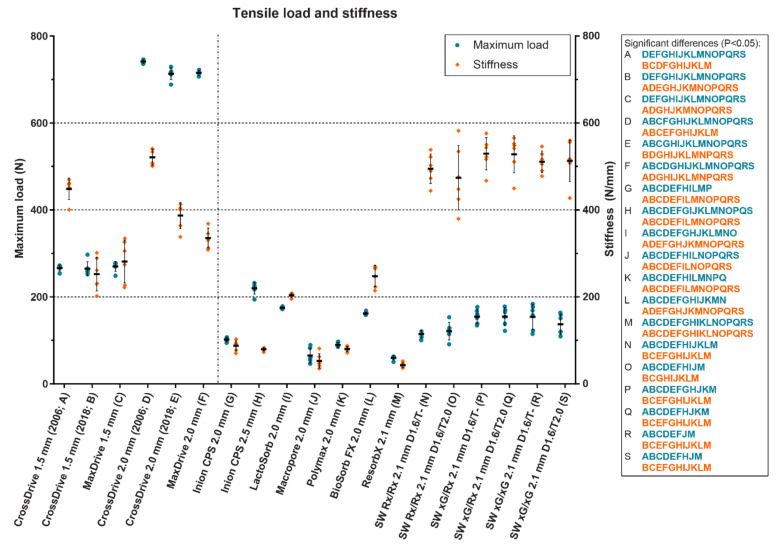
The tensile load and stiffness of 13 biodegradable and 6 titanium osteosynthesis systems commonly used in oral and maxillofacial surgery. The characters in blue and orange represent significant pairwise differences in maximum load and stiffness, respectively, between the corresponding systems using a one-way analysis of variance adjusted for multiple testing. The titanium CrossDrive (2006) plates consisted of 100% titanium produced by stamping of plates. The titanium CrossDrive (2018) and MaxDrive consisted of 100% titanium produced by milling of plates. The titanium CrossDrive (2006 and 2018) and MaxDrive screws consisted of a Ti6Al4V alloy. The composition of each biodegradable system is described in Table 1. Error bars: mean values  ±  standard deviation. Ti6Al4V, 90% titanium, 6% aluminum and 4% vanadium alloy; SW, SonicWeld; D, drill diameter (mm); T, tap diameter (mm). The dotted line separates the titanium (left) and biodegradable systems (right). Reprinted with permission from [20].

**Figure 8:**
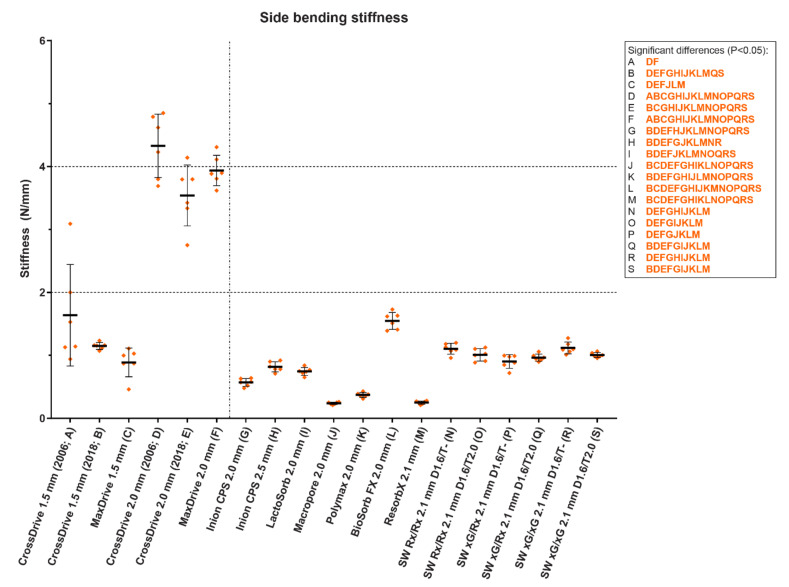
The side bending stiffness of 13 biodegradable and 6 titanium osteosynthesis systems commonly used in oral and maxillofacial surgery. The characters in blue and orange represent significant pairwise differences in maximum load and stiffness, respectively, between the corresponding systems using a one-way analysis of variance adjusted for multiple testing. The titanium CrossDrive (2006) plates consisted of 100% titanium produced by stamping of plates. The titanium CrossDrive (2018) and MaxDrive consisted of 100% titanium produced by milling of plates. The titanium CrossDrive (2006 and 2018) and MaxDrive screws consisted of a Ti6Al4V alloy. The composition of each biodegradable system is described in Table 1. Error bars: mean values  ±  standard deviation. Ti6Al4V, 90% titanium, 6% aluminum and 4% vanadium alloy; SW, SonicWeld; D, drill diameter (mm); T, tap diameter (mm). The dotted line separates the titanium (left) and biodegradable systems (right). Reprinted with permission from [20].

**Figure 9:**
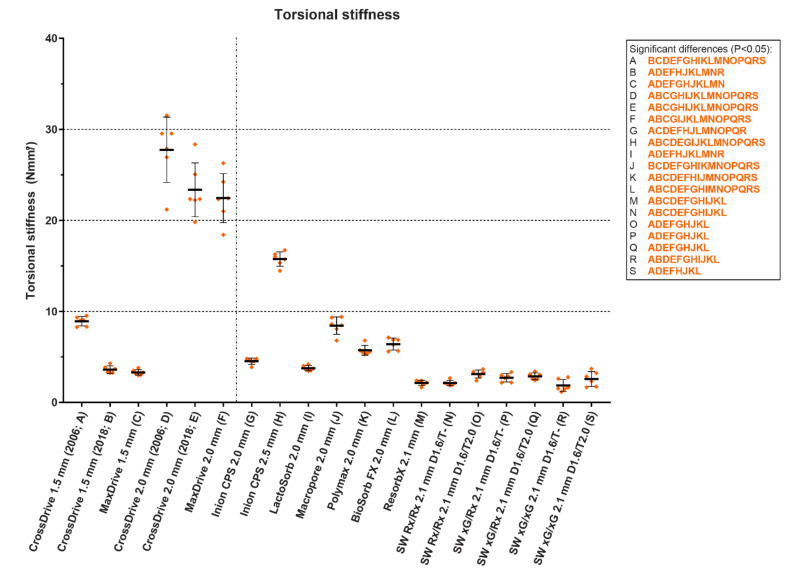
The torsional stiffness of 13 biodegradable and 6 titanium osteosynthesis systems commonly used in oral and maxillofacial surgery. The characters in blue and orange represent significant pairwise differences in maximum load and stiffness, respectively, between the corresponding systems using a one-way analysis of variance adjusted for multiple testing. The titanium CrossDrive (2006) plates consisted of 100% titanium produced by stamping of plates. The titanium CrossDrive (2018) and MaxDrive consisted of 100% titanium produced by milling of plates. The titanium CrossDrive (2006 and 2018) and MaxDrive screws consisted of a Ti6Al4V alloy. The composition of each biodegradable system is described in Table 1. Error bars: mean values  ±  standard deviation. Ti6Al4V, 90% titanium, 6% aluminum and 4% vanadium alloy; SW, SonicWeld; D, drill diameter (mm); T, tap diameter (mm). The dotted line separates the titanium (left) and biodegradable systems (right). Reprinted with permission from [20].

In a recent in vitro study, the maximum tensile load as well as the tensile, bending and torsional stiffness of 13 biodegradable and 6 titanium straight, four-hole osteosynthesis systems derived from static mechanical tests of the initial materials were assessed and compared (Figure 7, Figure 8 and Figure 9) [20]. The titanium systems’ tensile loads were higher than those of the biodegradable systems. The bending stiffness of the 1.5 mm titanium systems was comparable to all the biodegradable systems whereas the 2.0 mm system’s bending stiffness was higher. Regarding the biodegradable systems, Inion CPS 2.5 mm had the highest tensile load and torsional stiffness, SonicWeld 2.1 mm the highest tensile stiffness, and BioSorbFX 2.0 mm the highest bending stiffness. Regarding the titanium systems, the CrossDrive (2006) systems had the highest tensile, bending and torsional stiffness. It must be noted, though, that although high mechanical osteosynthesis properties are sought for adequate fixation, the extreme stiffness of the titanium systems can be a disadvantage due to the stress shielding of the underlying bone [12]. Stress shielding occurs when the underlying bone is exposed to less stress than it should endure, leading to an increase in osteoclast activity and bone resorption, that can, in turn, lead to decreased bone density and aseptic loosening [98,181]. This has led to the development of new titanium osteosynthesis systems with a lower elastic modulus to reduce stress shielding of the underlying bone by adjusting the production process (Figure 7, Figure 8 and Figure 9 and Table 3) [20,23,24].

Within the limitations of finite element analyses (e.g., assuming the masticatory forces are fixed), three-dimensional analyses indicated that the biomechanical stresses surrounding osteosynthesis systems remain far below the threshold of their ultimate strength of both biodegradable and titanium osteosynthesis systems [163,172,182,183]. In addition, the empirical evidence of fracture [19] and osteotomy [184] osteosyntheses shows that the efficacy of titanium and biodegradable osteosyntheses is similar (e.g., absence of malunion), indicating that the less favorable mechanical properties of biodegradable osteosynthesis are still sufficient to achieve similar healing outcomes. However, as also observed from the empirical evidence, the mechanical properties of biodegradable osteosyntheses of mandibular osteotomies may be insufficient to avoid micromovements [184]. Future research should also focus on these micromovements since they play an important role in developing foreign body reactions [26].

Finite element analyses also demonstrated that the stress surrounding conventional screws is much larger compared to those of plates, indicating that material complications may arise from the screws rather than the plates (e.g., screw loosening or fractures) [182]. The positive effect of ultrasound welding of biodegradable, thermoplastic pins instead of using conventional screws was demonstrated by the superior mechanical properties of the SonicWeld Rx (PDLLA with thermoplastic pins) compared to the Resorb X system (identical system with screws) [20]. Additionally, ultrasound welding caused a shift of the weakest link of the complete osteosynthesis system from the screw-plate interface to the plate itself. Therefore, ultrasound welding may reduce screw-related material complications, but this has to be investigated by future research.

## 3. Clinical Evidence

### 3.1. Biodegradable Versus Titanium Osteosyntheses: Efficacy and Symptomatic Removal

In theory, the primary advantage of biodegradable compared to titanium osteosynthesis is the reduced, or even eliminated, need for symptomatic device removal while having similar efficacy (i.e., adequate bone healing without malunion). In a recent systematic review with meta-analyses, the available clinical evidence (i.e., randomized controlled trials [RCTs], and prospective and retrospective controlled studies without language or period restrictions) from patients treated for maxillofacial fractures (i.e., Le Fort I, cranial, zygomaticomaxillary complex and mandibular fractures) with load-sharing biodegradable versus titanium osteosyntheses was compared [19]. Following a sensitive and thorough literature search that focused on all relevant clinical endpoints, the meta-analysis of RCTs demonstrated similar efficacy and morbidity between the two systems, but symptomatic osteosynthesis removal was significantly lower in the biodegradable compared to the titanium group [19]. Other studies that focused on cohorts of patients who had undergone load-sharing biodegradable fixation of midface and malar fractures [66,185,186], naso-orbital-ethmoid fractures [187], and mandibular body, parasymphysis and symphysis fractures [37,66,183], also showed adequate internal fixation, long-term stability, and biocompatibility with biodegradable systems. Similar results were observed in non-comparative cohorts that included adults with isolated orbital floor fractures [186] and combined orbital floor and medial wall fractures [187] treated with biodegradable mesh plates. In addition, another systematic review that focused on the composite of the complications (e.g., infection, palpability, dehiscence, material-related complications, and exposure) encountered with biodegradable versus titanium osteosynthesis of zygomatic and mandibular fractures revealed significantly fewer complications from biodegradable compared to titanium osteosyntheses, although the analysis had a risk of bias due to substantial methodological heterogeneity of the included studies [188]. Symptomatic biodegradable osteosynthesis system removal in these studies (0 to 8.6%) was comparable to that of the patients with mandibular, Le Fort I, and zygomatic fractures in a multicenter randomized controlled trial (RCT) that compared titanium (KLS Martin CrossDrive system) with biodegradable osteosynthesis (Inion CPS system) with a median follow-up of 99 (78;113) months [8].

Similar to the abovementioned results, a recent systematic review with meta-analyses that compared biodegradable versus titanium osteosyntheses in adults with dentofacial deformities treated with orthognathic surgery (i.e., Le Fort I, bilateral sagittal split (BSSO), and intraoral vertical ramus osteotomies (IVRO), with and without concurrent genioplasty) also demonstrated similar efficacy and morbidity between biodegradable and titanium osteosynthesis systems [184]. Non-comparative cohort studies that focused on the stability of maxillary [189,190,191], mandibular [189,190,191], or bimaxillary osteotomies [189,190,191,192,193], and post-operative complications [191,194,195], also showed predictable skeletal stability with post-operative complications rates similar to those reported in the literature after titanium osteosyntheses [196,197,198].

In contrast to the abovementioned trauma population [19], a non-significant difference in symptomatic biodegradable and titanium osteosynthesis system removal was observed in the aforementioned orthognathic population [184]. The main reasons for biodegradable osteosynthesis removal in both populations were chronic inflammation and discomfort [19,184]. Similar results were observed in the aforementioned RCT with long-term follow-up, but in-depth analyses revealed that all the removals among the biodegradable group were due to clinical problems in the mandible, and were only seen after fixation of osteotomies [8]. Different reviews noted that mandibular osteotomies are associated with significantly more complications and higher symptomatic biodegradable and titanium osteosyntheses removal compared to maxillary osteotomies, and compared to fracture fixation [188,199,200,201]. A large retrospective cohort study (n = 685 patients) that focused on the efficacy and complications of biodegradable osteosyntheses and symptomatic removal of the systems also showed that mandibular osteotomies are associated with more complications and higher symptomatic removal rates compared to other osteotomies [202]. In addition, earlier reviews also showed more complications and symptomatic osteosynthesis removal after titanium fixation of mandibular osteotomies compared to other osteotomies [199,200]. Together, these results indicate that biodegradable osteosynthesis is a viable alternative to titanium osteosynthesis for fixation of both fractures and osteotomies. However, among the studied trauma population, the symptomatic biodegradable osteosynthesis systems removal rates are lower compared to the titanium osteosyntheses group whereas the biodegradable and titanium osteosyntheses groups have similar symptomatic osteosyntheses removal rates after orthognathic surgery.

An important aspect of successful biodegradable osteosyntheses (i.e., adequate bone healing and stability, and lack of foreign body reactions) is the biomechanical perspective. The biomechanical differences between fixation of fractures and osteotomies may explain the beneficial effect of biodegradable systems compared to titanium systems after fracture fixation versus osteotomy fixation. Fracture fixation with interfragmentary stability ensures load-sharing osteosyntheses whereas in osteotomies, interfragmentary stability is absent and thus the complete load at the osteotomy is carried by the osteosynthesis system (i.e., load-bearing osteosyntheses) [1,203]. Since the mandible is exposed to considerably higher biomechanical forces compared to the maxilla, this effect is even more pronounced in mandibular osteosyntheses. This is supported by the empirical findings that symptomatic osteosynthesis removal after fixation of mandibular fractures and osteotomies is significantly higher compared to those of other parts of the facial skeleton, both after titanium and biodegradable osteosyntheses [188,199,200,201,202,204]. Biomechanical forces that are not sufficiently counteracted can result in micromovements surrounding the osteosyntheses that may result in disturbed bone healing or foreign body reactions [1,18,26,205,206,207,208]. Animal studies have shown that these micromovements should be limited to 28–150 µm to avoid fibrosis and accompanying foreign body reactions [26,205,206,207,208]. Therefore, it is important that the mechanical properties of biodegradable implants are sufficient for the intended clinical application to ensure (primary) stability for adequate bone healing and to avoid micromovements and the accompanying risk of foreign body reactions (Table 2).

The clinical evidence of biodegradable metals is limited. A clinical study that compared magnesium (MgYREZr alloy; WE43) with titanium compression screws (Ti6Al7Nb alloy) for the fixation of mandibular condylar head fractures showed similar results after 18-month follow-up, with limited formation of hydrogen gas within the first 4 months [86]. These and other intermediate-term clinical outcomes [209] indicate that tuning the composition and structure of magnesium alloys are promising techniques to achieve biodegradable metal osteosyntheses in maxillofacial surgery. Although the degradation profiles of biodegradable magnesium alloys look promising [210,211,212], future research should focus on in vivo studies with long-term follow-up.

### 3.2. Biodegradable Versus Titanium Osteosyntheses: Secondary Advantages

At first glance, the non-significant difference in the proportion of symptomatic removal of biodegradable and titanium osteosyntheses in orthognathic surgery seems to negate the benefits of biodegradable osteosyntheses. However, asymptomatic biodegradable osteosynthesis systems will eventually be resorbed while titanium osteosynthesis will remain in situ until removed surgically. Therefore, titanium osteosynthesis systems have a life-time risk of, e.g., late infection or palpability complaints. Furthermore, biodegradable osteosyntheses have other (secondary) advantages, besides the most obvious benefit of less device removal compared to titanium systems, including no interference with radiographic imaging and radiotherapy, a more gradual transfer of stress to the healing bone (i.e., less stress shielding), and less system palpability in the long-term [8,9,19,176,213,214,215]. Whereas 17–80% of the patients undergo a second operation for elective titanium osteosynthesis removal due to their awareness of the presence of a foreign body (i.e., the titanium system) [216,217,218], asymptomatic biodegradable systems are generally eventually resorbed, thus forestalling such elective removals. When asked prior to surgery, the vast majority of patients (i.e., >95%), therefore, prefer biodegradable over titanium osteosyntheses in both maxillofacial traumatology and orthognathic surgery [219,220]. Despite the similar symptomatic removal rates in orthognathic surgery, the other benefits of biodegradable over titanium systems (e.g., no temperature sensitivity, no possible growth restrictions, and no hampering of imaging and radiotherapy) could also be valid reasons to choose biodegradable osteosynthesis systems. All these aspects should therefore be addressed preoperatively when informing the patients to ensure well-informed decision making.

Similarly, after fracture fixation in pediatric patients, common practice is to electively remove all the titanium systems due to possible later growth disturbances [221,222] and plate migration [223,224,225], whereas only the symptomatic biodegradable systems (12%) are removed [19]. In a recent systematic review including pediatric upper- and mid-facial fractures, the biodegradable osteosyntheses showed significantly fewer complications and symptomatic osteosynthesis system removal rates compared to titanium osteosyntheses [226]. Comparable rates were observed after applying biodegradable osteosynthesis for mandibular fracture fixation [227,228] and in craniofacial surgery [229,230] in pediatric patients. Biodegradable osteosyntheses would thus overall result in lower osteosynthesis removal compared to titanium osteosyntheses from pediatric patients. It must be noted, though, that there is currently no conclusive evidence whether growth disturbances actually occur with titanium systems and, thus, there is controversy in the current literature regarding the elective removal of titanium osteosynthesis systems from pediatric fracture patients [223,224,231,232]. Therefore, this subject needs to be addressed by future research. OMF-surgeons should inform pediatric patients and/or their caregivers about the above preoperatively to guide the shared-decision making.

### 3.3. Certainty of the Current Evidence

An important aspect when assessing efficacy, morbidity and symptomatic osteosynthesis removal in clinical studies is the duration of follow-up. The studies included in the most recent and comprehensive systematic reviews that focused on biodegradable versus titanium osteosyntheses in maxillofacial traumatology [19] and orthognathic surgery [184] predominately had 2-year follow-ups. Although this is sufficient follow-up to assess efficacy (e.g., adequate bone healing), symptomatic osteosynthesis removal can occur later on (i.e., >2-year follow-up) due to osteosynthesis system palpability, thermal sensitivity or foreign body reactions [8,53,65,69,233,234,235]. In a cohort study with a follow-up of 67 months, 7% of the included patients underwent symptomatic biodegradable osteosynthesis removal between 24 and 67 months [186]. In an RCT with a follow-up of 99 months, 3% and 5% of the patients in the biodegradable and titanium group, respectively, underwent a secondary surgical procedure to remove symptomatic osteosynthesis system removal between the 24-month and final follow-up [8]. Similarly, titanium system removals due to infection and discomfort occurred after the 4- [201] and 5.5-year follow-ups [235]. Therefore, future research should also include long-term follow-up assessments (e.g., ≥5-year follow-ups) in the pre-specified protocols.

Since significant differences in biocompatibility and degradation profiles as well as in mechanical properties are observed between copolymeric biodegradable osteosynthesis systems, future clinical research should focus on a specific biodegradable system that has proven to be biocompatible in the long-term as well as having the most favorable mechanical properties for specific surgical indications. In addition, there is currently no evidence to support or refute the use of biodegradable osteosynthesis in load-bearing fracture fixation. Since current evidence suggests that load-sharing fixation of fractures using biodegradable osteosynthesis is feasible, the next step would be to focus on load-bearing biodegradable osteosynthesis of fractures. Finally, systematic reviews should include tools to assess the degree of clinical heterogeneity [236], and should perform network meta-analyses to assess the most preferred biodegradable system. This would allow conclusions regarding the efficacy of specific biodegradable osteosynthesis systems based on clinical evidence.

## 4. Clinical Recommendations: Titanium or Biodegradable Osteosyntheses?

Current pre-clinical and clinical evidence indicates that biodegradable copolymeric osteosynthesis is a viable alternative to titanium osteosynthesis for fixation of both fractures and osteotomies, with similar efficacy [19,184]. Fixation of fractures also leads to significantly lower symptomatic device removal, thereby achieving the primary advantage of biodegradable osteosyntheses. Based on both the biological and biomechanical perspectives [20,25], a biodegradable osteosynthesis system composed of amorphous copolymers (e.g., PDLLA), preferably using ultrasound welding with a well-contoured shape without acute angles, and that has sufficient mechanical properties has the greatest potential as a biocompatible biodegradable copolymeric osteosynthesis system. Therefore, for midface fractures, the SonicWeld Rx 2.1 mm system is recommended [20,25]. The Inion CPS 2.5 mm system is recommended for mandibular fractures. Whenever the patient or surgeon prefers a titanium osteosynthesis system, 1.5 (e.g., midface fractures) and 2.0 mm titanium systems (e.g., mandibular fractures) from several suppliers are recommended [1,20,170].

Although copolymeric biodegradable and titanium osteosyntheses of osteotomies result in similar efficacy, both groups also have similar symptomatic osteosyntheses removal rates. Therefore, the primary advantage of biodegradable osteosyntheses in this population is neglected. Titanium systems are, therefore, still preferred due to lower risk of perioperative screw breakage, lower operation time, and better perioperative handling compared to biodegradable systems [19,171,184]. For maxillary and mandibular osteotomies, 1.5 and 2.0 mm titanium systems are recommended, respectively. Biodegradable osteosyntheses in orthognathic surgery may still be used, but only whenever the patient and/or surgeon prefer biodegradable over titanium systems, e.g., on the basis on secondary advantages (e.g., no temperature sensitivity, no possible growth restrictions, and no hampering of imaging). Then, the SonicWeld Rx 2.1 mm and Inion CPS 2.5 mm systems are also recommended for maxillary and mandibular osteotomies [20,25], respectively.

There is currently no evidence to support or refute the use of biodegradable osteosynthesis in load-bearing fracture fixation (e.g., a comminuted fracture) or osteosyntheses in reconstructive surgery (e.g., anatomical defects after an oncological resection) [19]. However, since the biomechanical requirements for these surgical procedures are comparable or even higher compared to osteosyntheses of osteotomies [1,20,166], titanium osteosyntheses remain preferred over biodegradable osteosyntheses until (empirical) evidence becomes available that supports the use of biodegradable osteosyntheses for these surgical procedures.

## 5. Future Perspectives

### 5.1. Overcoming the Disadvantages of Current Osteosynthesis Systems

#### 5.1.1. Biodegradable (Co)Polymeric Systems

In Table 2, different important aspects of biodegradable osteosynthesis systems have been summarized accompanied with the ideal properties and the potential solutions to accomplish these properties.

Both in maxillofacial trauma treatment and orthognathic surgery, the risk of perioperative screw breakage and perioperative time is significantly higher in the biodegradable compared to titanium osteosyntheses group [19,184]. Perioperative biodegradable screw breakage is a commonly reported complaint [237,238] and, together with the need to pre-tap burr holes for biodegradable screws (i.e., a time-consuming extra step), it is the lowest rated perioperative handling aspect of biodegradable systems by OMF-surgeons [238].

Screw breakage is more likely when the difference between the torque applied to the screws for adequate fixation (i.e., hand-tight) and the maximum allowed torque (i.e., torque up to screw breakage) is small [239,240]. The difference between hand-tight and maximum torque is much smaller for biodegradable screws composed of synthetic polymers compared to titanium screws [20,239,240] and, thus, explains the higher risk of perioperative biodegradable screw breakage. Biodegradable screws composed of degradable metals or silk have more better mechanical properties that can be prepared as self-tapping screws as well as that screw breakage occurs less often than when using screws of synthetic materials [94,209].

An alternative to biodegradable screws is biodegradable pins that are inserted via ultrasonic welding (e.g., the SonicWeld systems), thereby diminishing the risk of perioperative screw breakage (Table 2). This also obviates the need to pre-tap the burr holes. Ultrasound welding has been shown to be easy to use and reduces the time needed to apply the osteosynthesis systems by up to 50% compared to the same biodegradable system with screws [229,230,241].

Besides the advantages in perioperative handling, systems with ultrasound pin welding have significantly better mechanical properties compared to an identical system with conventional screws [20,176]. A positive effect of ultrasound welding was demonstrated by the superior mechanical properties of the SonicWeld Rx (PDLLA with thermoplastic pins) compared to the Resorb X system (identical system with screws; Figure 7, Figure 8 and Figure 9) [20].

Improvements in mechanical properties may also result in smaller biodegradable osteosynthesis devices, thereby reducing issues regarding palpability of the system by patients, and stress-free closure of the incision by OMF-surgeons. In addition, low implant volume reduces the amount of acidic degradation products and thus reduces the risk of (late) foreign body reactions [18]. Studies have also shown that the geometry of the implant also affects the host response. A smooth, well-contoured shape without acute angles induces macrophage polarization towards M2 macrophages (i.e., towards wound repair and an immune regulatory phenotype) whereas implants with acute angles and non-contoured shapes increase the risk of foreign body reactions to biomaterials [25,79,80]. Since screws possess acute angles and welded pins are smooth without acute angles, welded pins may also contribute to a more biocompatible osteosynthesis system compared to a similar system with screws [18].

Besides ultrasound welding, patient-specific osteosynthesis systems will also contribute to osteosynthesis systems with superior mechanical properties while reducing the total volume of the system, i.e., the system can be strengthened where necessary while removing excess material [18,83,242,243]. This will also reduce the amount of degradation products and, thus, the risk of foreign body reactions [18].

Other novel developments such as different types of coatings to prevent bacterial adhesion, surface topography adjustments to eliminate surrounding bacteria without the need for antibiotics, and coatings with antibiotics that can be released locally using ultrasound are promising developments to reduce infection risk (Table 2). These leads should be included in future research.

#### 5.1.2. Titanium Systems

In Table 3, different important aspects of titanium osteosynthesis systems have been summarized accompanied with the ideal properties and the potential solutions to accomplish these properties.

Titanium osteosynthesis systems have been improved to overcome the associated disadvantages. The titanium alloy production process can be altered in such a way to increase or decrease the elastic modulus of the titanium osteosynthesis plates [23,24]. By increasing the elastic modulus, the titanium plates can be thinner while maintaining sufficient mechanical properties for adequate bone healing. Another advantage of thinner systems is that they may reduce the tactile sensation of the osteosynthesis systems for patients which, in turn, could reduce symptomatic osteosynthesis removal rates. In addition, reducing the volume of the titanium osteosynthesis systems reduces imaging and radiotherapy restrictions. On the other hand, decreasing the elastic modulus of existing osteosynthesis plates would address the potential issue of stress shielding of the underlying bone [23,24] as well as improve the perioperative handling of the systems (Table 3).

Besides the adjustments in mechanical properties, different types of surface modifications and coatings have been introduced to decrease infection risk, improve osteogenesis surrounding the implant, and reduce wearing of particles [26,110]. Surface modifications, such as adjusting the nano-scale surface topography (e.g., pillars on the surface) [84], can lead to the elimination of surrounding bacteria. Another promising surface modification is oxygen plasma immersion ion implantation. Here, the implant surface is modified by targeting it with specific ions (e.g., TiO_2_) to gain specific properties including inhibiting various bacteria from adhering (e.g., *Staphylococcus aureus* and *Escherichia coli*) and reducing metal ion release from the implant [110]. Finally, different types of coatings have also been introduced to prevent bacterial adhesion to implant surfaces, diminishing the need for antibiotics (Figure 6 and Table 3) [26].

### 5.2. Outlook

Besides the currently available biodegradable synthetic copolymeric systems (e.g., PDLLA), novel biodegradable systems composed of degradable metals (e.g., magnesium and zinc alloys) [87,244,245] or natural polymers (e.g., amorphous silk fibers derived from the silkworm *Bombyx mori*) are being developed [18].

Biodegradable metals are promising alternatives to polymeric osteosynthesis but a major challenge of biodegradable metals is the unpredictable degradation profile in vivo [18]. Therefore, although the short-term degradation profiles of biodegradable magnesium and zinc alloys look promising [210,211,212], research should focus on controlling the degradation rates in vivo and assessing the long-term outcomes of biodegradable metal systems in a clinical setting.

Silk is the most recent addition to biodegradable materials for osteosynthesis systems [18]. The preliminary fracture fixation results are promising [94]. However, no data are currently available on the short- and long-term effects of in vivo produced degradation products. Future research should focus on these aspects as well as on the degradation pathways in vivo and the comparison of the biocompatibility and safety profiles with other available biodegradable materials.

Besides material composition, the microstructure of biomaterials, material morphology, geometry, internal structure, surface topology, porosity, and coatings require more attention as these are important factors that contribute to the host response (Table 2 and Table 3) [18,246]. Surface modifications (e.g., polarity and charge) have been shown to influence cellular behaviour [247,248,249,250] and that surface coatings can have antimicrobial effects [85]. Tuning the spherical dimensions of biomaterials increases their biocompatibility [72]. Furthermore, bioactive molecules can be incorporated into biomaterials [251] which may be useful for incorporating antibiotics into osteosynthesis systems used in revision surgery following an infection. These factors may improve the next-generation (biodegradable) osteosynthesis systems so that the host responses are influenced and the risk of surgical site infections is decreased (Table 2) [72].

Research should also aim to assess the least required mechanical properties for an osteosynthesis system for specific cases (e.g., by developing in silico models). Ideally, these insights should be incorporated into a validated model in which osteotomy and fracture parameters (e.g., presence of interfragmentary stability, gap width, and mandibular height) can be easily adjusted. In addition, three-dimensional printing technologies such as stereolithography and selective laser sintering [252] means that patient-specific biodegradable osteosynthesis systems are now feasible [83,253]. Applying such new design methods could lead to implants with a better fit, stress resistance and dimensions (Table 2 and Table 3) [26]. An excellent example is a patient-specific osteoinductive implant made by stereolithography to repair orbital floor defects that has shown promising results [83]. Constructing and validating in silico models would also contribute to, and accelerate, the translation to patient-specific biodegradable osteosyntheses systems for maxillary and/or mandibular fractures and osteotomies [18].

## 6. Conclusions

In this review, the current literature that compared the in vitro and in vivo performances (i.e., including the clinical performances) of different biodegradable and titanium osteosynthesis systems was discussed. It was shown that, based on current pre-clinical and clinical evidence, biodegradable copolymeric osteosyntheses are a viable alternative to titanium osteosyntheses when applied to treat maxillofacial trauma, with similar efficacy and significantly lower symptomatic osteosynthesis removal, but with higher perioperative screw breakage. For orthognathic surgery, biodegradable copolymeric osteosynthesis is also a valid alternative to titanium osteosyntheses, but with longer operation times compared to titanium osteosyntheses. Furthermore, it was shown that an osteosynthesis system composed of an amorphous copolymer (e.g., PDLLA), preferably using ultrasound welding with a well-contoured shape without acute angles, and that has sufficient mechanical properties (e.g., the SonicWeld Rx 2.1 mm system) has the greatest potential as a biocompatible biodegradable copolymeric osteosynthesis system. Future research should focus on surface modifications (e.g., nanogel coatings and surface topography) of titanium and biodegradable osteosynthesis systems to improve surgical handling, osteogenesis, and wear resistance. Finally, novel biodegradable materials (e.g., magnesium alloys and silk) are promising candidates for the development of next-generation biodegradable osteosynthesis systems.
